# Positive Effect of Human Milk Feeding during NICU Hospitalization on 24 Month Neurodevelopment of Very Low Birth Weight Infants: An Italian Cohort Study

**DOI:** 10.1371/journal.pone.0116552

**Published:** 2015-01-15

**Authors:** Dino Gibertoni, Luigi Corvaglia, Silvia Vandini, Paola Rucci, Silvia Savini, Rosina Alessandroni, Alessandra Sansavini, Maria Pia Fantini, Giacomo Faldella

**Affiliations:** 1 Department of Biomedical and Neuromotor Sciences, Unit of Hygiene and Biostatistics—University of Bologna, Bologna, Italy; 2 Neonatology and Neonatal Intensive Care Unit—S. Orsola-Malpighi Hospital, Department of Medical and Surgical Sciences, University of Bologna, Bologna, Italy; 3 Department of Psychology, University of Bologna, Bologna, Italy; TNO, NETHERLANDS

## Abstract

The aim of this study was to determine the effect of human milk feeding during NICU hospitalization on neurodevelopment at 24 months of corrected age in very low birth weight infants. A cohort of 316 very low birth weight newborns (weight ≤ 1500 g) was prospectively enrolled in a follow-up program on admission to the Neonatal Intensive Care Unit of S. Orsola Hospital, Bologna, Italy, from January 2005 to June 2011. Neurodevelopment was evaluated at 24 months corrected age using the Griffiths Mental Development Scale. The effect of human milk nutrition on neurodevelopment was first investigated using a multiple linear regression model, to adjust for the effects of gestational age, small for gestational age, complications at birth and during hospitalization, growth restriction at discharge and socio-economic status. Path analysis was then used to refine the multiple regression model, taking into account the relationships among predictors and their temporal sequence. Human milk feeding during NICU hospitalization and higher socio-economic status were associated with better neurodevelopment at 24 months in both models. In the path analysis model intraventricular hemorrhage—periventricular leukomalacia and growth restriction at discharge proved to be directly and independently associated with poorer neurodevelopment. Gestational age and growth restriction at birth had indirect significant effects on neurodevelopment, which were mediated by complications that occurred at birth and during hospitalization, growth restriction at discharge and type of feeding. In conclusion, our findings suggest that mother’s human milk feeding during hospitalization can be encouraged because it may improve neurodevelopment at 24 months corrected age.

## Introduction

Neurodevelopment of infants born prematurely has been receiving a growing attention in the last decades. Several longitudinal cohort studies [1–3] reported that preterm infants are at higher risk of long-term disability and cognitive impairment than term infants. Many factors including feeding strategies could affect the development and function of the brain [4].

A high-energy and nutrient intake is currently recommended for preterm newborns in the attempt to achieve a postnatal growth that mirrors a fetus of equivalent postmenstrual age [5, 6]; this should be ideally achieved by means of fortified human milk [7]. Although a high protein and energy nutritional regimen leads to better weight growth and also to catch-up growth in head circumference [8], its effect on long-term neurodevelopment is controversial [9–11]. A recent randomized clinical trial showed that neurodevelopment at 24 months is similar between newborns fed nutrient-enriched formula and those fed standard full-term formula for 6 months corrected age (CA) [12]. However, during the initial weeks of life a full nutritional support is difficult in very low birth weight (VLBW) infants, due to the poor feeding tolerance related to gastro-intestinal immaturity. Thus, a relevant deficit in energy and nutrients is accrued during NICU stay and many preterm infants are growth restricted at discharge [13–15]. Evidence from studies about growth and development is controversial: some studies suggest that better growth in weight and head circumference has a positive effect on development, probably mediated by a better brain growth and neurological maturation [14–17], other studies report that the positive effect of human milk feeding overcomes the delayed weight gain associated to the lower protein and energy intake of human milk compared with formula milk [18, 19].

In order to elucidate the complex relationship linking nutrition with neurodevelopmental outcomes, we examined the impact of human milk feeding during hospitalization on 24 month CA neurodevelopment in two ways. First, we measured the effect of human milk feeding on neurodevelopment after adjusting for the effects of perinatal and early postnatal factors using a multiple linear regression model. Second, we developed a path analysis model that takes into account the temporal sequence of the factors examined and their mutual relationships.

## Materials and Methods

### Study population

The study cohort includes all VLBW infants (weight <1500 g) and/or infants born at ≤32 weeks of gestational age, admitted at birth to the Neonatal Intensive Care Unit (NICU) of S.Orsola University Hospital, Bologna (North Eastern Italy), from January 1^st^ 2005 to June 30^th^ 2011. Newborns were recruited in a follow-up program up to 24 months corrected age. Infants with severe congenital malformations were excluded from the study.

### Ethics statement

Written informed consent to participate in the study was obtained from the infants’ parents or legal guardians. Data were anonymized prior to data analysis and the study protocol (NEO-13–05) was approved by the Ethics Committee of the Azienda Ospedaliero-Universitaria of Bologna, Italy.

### Outcome variable

Neurodevelopment at 24 months CA was evaluated using the revised Griffiths Mental Development Scale 0–2 years [20]. This instrument is one of the most popular developmental test designed for use in children aged 0–2 years, particularly in the UK and Europe [21]. It requires approximately 45 min to be administered and evaluates five functioning domains: Locomotor, Personal-Social, Hearing and Language, Eye and Hand Coordination, Performance. It yields standardized domain scores and a composite General Quotient (GQ, Mean 100.5, SD 11.8). In this study, the test was administered by two psychologists with long-standing experience in developmental assessment.

The psychologists were aware of the aim of the study but they did not know nutritional data.

In line with previous studies [22, 23] the GQ was calculated using the tables of standardized scores for the English infants population [22], because the standardization for the Italian population is not available. For descriptive purposes the GQ was classified into the following categories: normal development (≥88.7), mild (88.6–76.9), moderate (76.8–65.1) and severe delay (≤65) [23] and in the path analysis it was centered around the median and divided by 10 to reduce its variance and obtain unbiased estimates. [24]

### Study variables

The main predictor of interest was feeding during hospitalization. Information on feeding was recorded at discharge and coded as human milk (own mother’s raw milk, either given by bottle or directly from the breast), mixed (human milk for more than 50% of the daily intake) or exclusive formula milk. We assumed that infants fed human milk at discharge, either exclusive or mixed, had this type of feeding for the entire period of hospitalization. Preterms fed formula milk at discharge were either those fed formula milk during all their hospitalization or those that switched from human milk to formula milk during hospitalization. Information on the day of switch from human to formula milk was not recorded.

The in-hospital feeding protocol prioritizes human milk when possible. Newborns are breastfed, fed fortified expressed breast milk, mixed or formula, according to the available quote of human milk. As soon as possible, babies start breastfeeding; the portion of human milk taken by breastfeeding is not fortified. Fortification of bottle-administered human milk is routinely done during hospitalization at standard dosage with a commercial preparation. It starts when daily enteral intake reached 100 ml/Kg and is recommended after discharge until the weight of 3.5 kg was achieved. When needed, “preterm formula” containing 80–90 kcal/100 ml and proteins 2–2.3g/100 ml [25] is used during hospitalization and “post-discharge formula” containing 72–74 Kcal/100ml and proteins 1.8–1.9 g/100 ml is recommended after discharge until the weight of 3.5 kg was achieved.

The covariates included in the analyses were biological characteristics or complications occurred at birth or during hospitalization, that were chosen a priori based on their clinical significance and evidence from the literature of an association with neurodevelopment. Gestational age (GA) in weeks was based on the last menstrual period and first-trimester scan.

Weight (in grams) of the infants was collected at birth (BW) and at discharge by a trained neonatologist, and was measured twice at each time to reduce possible errors. Weight Z-scores were calculated using the Italian Neonatal Anthropometric Charts. [26] that were developed for newborns up to 42 weeks CA. For the few infants discharged at CA>42 weeks, weight Z-score at discharge was calculated using the Eurogrowth reference that were developed for term newborns > = 40 weeks CA [27]. Italian charts were preferred because they have been recently published and are more suitable for our cohort. In the overlapping period of 40 to 42 weeks CA, Italian and Eurogrowth charts are comparable, therefore we do not expect to introduce a bias by using two different reference charts. Weight measures were then recoded into binary indicators that classify as growth restricted at birth (SGA) and at discharge (EUGR) those infants with a standardized weight <-1.28, corresponding to the 10^th^ percentile of the distribution.

Interventions and comorbidities observed during hospitalization were mechanical ventilation (MV), chronic lung disease (BPD, oxygen need at 36 weeks postmenstrual age), early and late onset sepsis (including both culture proven or clinical sepsis), necrotizing enterocolitis (NEC, requiring surgery), severe intra-ventricular hemorrhage (grade 3 and 4 as classified by Papile et al., [28] including post-hemorrhagic hydrocephalus requiring surgery) or periventricular leukomalacia, classified as the presence of periventricular cysts at any cranial ultrasound performed during hospital stay (IVH-PLV). Severe rethinopathy of prematurity (stages 3 to 5 according to the International Committee for the Classification of Retinopathy of Prematurity [29]) was not considered in the analyses because it had a very low prevalence (only 5 newborns). Socio-economic status (SES) was measured using the Hollingshead Index; [30] this index is obtained by combining the information on education and occupation of newborns’ parents and ranges from 0 to 66. For the analyses, GA was centered around the median and SES was centered around the median and divided by 10.

### Statistical analysis

Descriptive statistics included mean, median and standard deviation for continuous variables and frequencies for categorical variables. The effect of human milk feeding on neurodevelopment was examined first using a univariate linear regression. Then in multivariate models the effect of feeding was adjusted first for complications and second for all covariates.

Path analysis (PA) was used to model the effect of human milk on neurodevelopment, taking into account the relationships among predictors and their temporal sequence. This analytical strategy has already been used in the neonatology field to investigate the determinants of neurodevelopment [31] and of chronic lung disease [32]. Path analysis has advantages over the more traditional multiple regression because it allows to test whether relationships among variables hypothesized a priori fit to the data. Moreover, PA incorporates procedures for the estimation of missing values of the outcome variable, while regression uses only subjects with complete data thereby reducing the sample size. Specifically, Full Information Maximum Likelihood (FIML) [33] was used to predict the missing values of the outcome. This method can be applied when data missingness is unrelated with the outcome [34].

We developed two different PA models, to take into account that the date of occurrence of complications and the date of switch from human milk feeding to formula are not available. Therefore, the temporal sequence of these events cannot be clearly established. In the first model we hypothesized that type of feeding and complications were unrelated, and in the second model we assumed that type of feeding depended on complications.

In the PA models predictors were arranged starting from GA and SGA, followed by complications and type of feeding during hospitalization and then by growth restriction. We hypothesized that neurodevelopment was directly influenced by each predictor and that the EUGR condition would be influenced by the presence of complications, by the type of feeding and by newborn characteristics recorded at birth (gestational age and weight restriction). Finally, complications and type of feeding were hypothesized as influenced by GA and weight restriction at birth; type of feeding was also considered as affected by SES. Starting from the initial models including all the hypothesized relations, final models were obtained by trimming all non-significant effects (p>0.05). All the effects in the PA model were measured by standardized regression coefficients. The goodness of fit to the data in the PA model was assessed using RMSEA, [35] CFI [36] and TLI [37] statistics and the proportion of variance of neurodevelopment explained by the predictors. A satisfactory goodness of fit is denoted by RMSEA values <0.05 [38] CFI and TLI values >0.90 [39].

Mplus 7.11 (Muthén & Muthén, Los Angeles, California, USA) was used for path analysis; all the other analyses were carried out using Stata 13.1 (StataCorp LP, College Station, Texas, USA).

## Results

From January 2005 to June 2011, 511 VLBW newborns were admitted at birth to the NICU (mean GA 29.0±2.7 weeks; mean BW 1179±373 g); 501 (98.0%) of them were inborn and 166 (32.5%) had BW<1000g. Forty (7.8%) died during hospitalization, two were excluded due to trisomy 21, two died during the follow-up program and two were excluded because they were hospitalized for at least 18 months due to short bowel syndrome. Newborns lost to follow-up were 146 (28.6%), mainly because they were living far from our center or for parents’ unavailability to attend visits. We further excluded from the analyses 3 preterms because they were outliers with at least four NICU complications and more than 150 days of hospitalization. Compared with the 146 newborns excluded, the 316 newborns included in the analyses had a significantly lower mean birthweight (1149.1 g vs. 1359.8 g; t-test: t = -6.24, p<0.001) and gestational age (29.0 wks, vs. 30.9 wks; t-test: t = -8.40, p<0.001) and significantly higher proportions of MV (25,3% vs. 15.5%; p = 0.019), sepsis (14.0% vs. 4.9%; p = 0.005) and BPD (20.9% vs. 2.8%, p<0.001) at the chi-square independence test.

Demographic and clinical characteristics of the study population are listed in Table 1. Newborns were 17.1% SGA and 62.3% EUGR. Feeding started as human or mixed milk for 300 newborns (94.9%), formula for 11 newborns (3.5%), while for the remaining 5 newborns (1.6%) data on initial feeding were not recorded. Feeding during hospitalization was 34.5% human milk (n = 109), 36.1% mixed (n = 114) and 29.4% formula milk (n = 93). Of the 300 starting with human milk feeding, 77 switched to formula milk (25.7%). The reasons for switching to formula feeding were mothers’ insufficient milk production (n = 34, 44.2%) or the need of special milk due to infants’ feeding intolerance (n = 39, 50.6%) and unknown in 4 newborns (5.2%). Of these 77 newborns, 58.4% (n = 45) had at least one complication. Among those fed human milk throughout the hospitalization, the percentage with at least one complications was 29.2% (65/223) and among those fed exclusively formula it was 45.4% (5/11). The χ²-test comparing the proportions of complications was equal to 21.43 (p<0.001), with a post-hoc significant difference only between those switching and those fed exclusively human milk (χ² = 21.15, p<0.001).

**Table 1:** Characteristics of the study sample.

**Variables**	**n**	**Frequency or mean±sd**	**% or median**
Female	316	153	48.4
Weight at birth (g)	316	1149.1±341.2	1176
Weight at discharge (g)	313	2110.6±386.7	1982
GA (weeks)	316	29.0±2.3	29
SGA	316	54	17.1
EUGR	313	195	62.3
Feeding at discharge	316		
Human milk		109	34.5
Mixed milk		114	36.1
Formula milk		93	29.4
Intraventricular haemorrhage or Periventricular leukomalacia	316	19	6.0
Retinopathy of prematurity	315	5	1.6
Mechanical ventilation	316	80	25.3
Chronic lung disease	315	66	21.0
Sepsis	315	44	14.0
Necrotizing enterocolitis	316	12	3.8
Socio-Economic Status	312	26.88±9.8	28
GQ at 24 months CA	276	93.4±15.8	97
mild delay		54	19.6
moderate delay		10	3.6
severe delay		21	7.6
Weight at 24 months CA	266	11563.4±1660.2	11607.5
Underweight at 24 months CA	266	87	32.7

Mean±SD GQ at 24 months of CA was 96.7±12.6 for infants fed exclusively human milk, 95.1±16.2 for infants fed human milk and formula, and 87.2±17.1 for infants fed exclusively formula milk at discharge; because the mean GQ was similar in newborns fed human or mixed milk, we used a dichotomous variable to characterize human vs. formula milk for further analyses.

GQ at 24 months CA was missing for 40 newborns (12.7%). For 37 of them the reason for drop-out was that parents moved to towns distant from our centre or had working problems that prevented them from attending the follow-up visits; only 3 newborns were lost to follow-up because their developmental impairment became too severe.

### Multiple regression analysis

In the univariate regression, that was performed on 276 cases because of missing values on neurodevelopment, the standardized regression coefficient for type of feeding was β = 0.247 (p<0.001). When complications were added to the model (n = 275), the coefficient for type of feeding changed to β = 0.159 (p = 0.004), suggesting that complications play a confounding role, but the effect of type of feeding remained significant. In the final multiple regression model (Table 2) adjusted for GA, SGA, SES, IVH/PVL, NEC, MV, sepsis and EUGR (n = 271), human milk retained a significant positive effect on neurodevelopment (β = 0.109, p = 0.050): infants fed human milk had an estimated 3.80 points higher GQ score compared to those formula fed. Two other variables had a significant relationship with neurodevelopment: IVH/PVL (β = -0.361, p<0.001) and SES (β = 0.203, p<0.001). Newborns with IVH/PVL had an estimated 23.31 points reduction in GQ score compared with newborns without the complication, while GQ score increased by 3.28 points for a 10 point increase of SES. Overall the percentage of GQ variance accounted by multiple regression was 31.3%.

**Table 2:** Results of the multiple linear regression of neurodevelopment on the complete set of predictors (n = 271, R^2^ = 0.313).

	**Coefficient**	**Beta**	**p**
Human milk feeding during NICU	3.799	0.109	0.050
IVH and/or PVL	-23.307	-0.361	<0.001
NEC	-5.067	-0.064	0.246
Sepsis	-1.124	-0.025	0.667
Mechanical Ventilation	-3.831	-0.107	0.108
Gestational age	0.810	0.124	0.069
SGA	1.432	0.034	0.546
EUGR	-1.408	-0.043	0.453
Socio-economic status	3.284	0.203	<0.001
Constant	94.488		<0.001

### Path analysis

**Figure 1:**
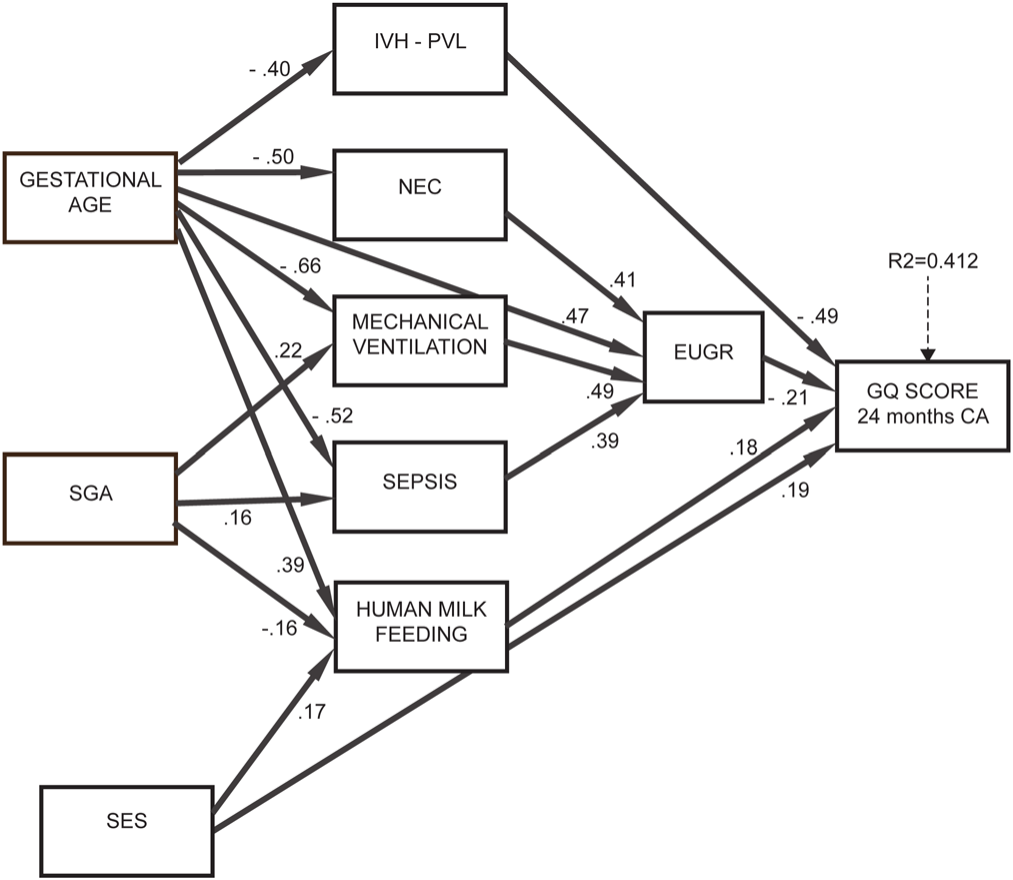
Results of path analysis, model 1. The numbers on the arrows are standardized regression coefficients that indicate the strength and direction of effects between variables. *R*² indicates the proportion of neurodevelopment variance explained by the predictors.

**Figure 2:**
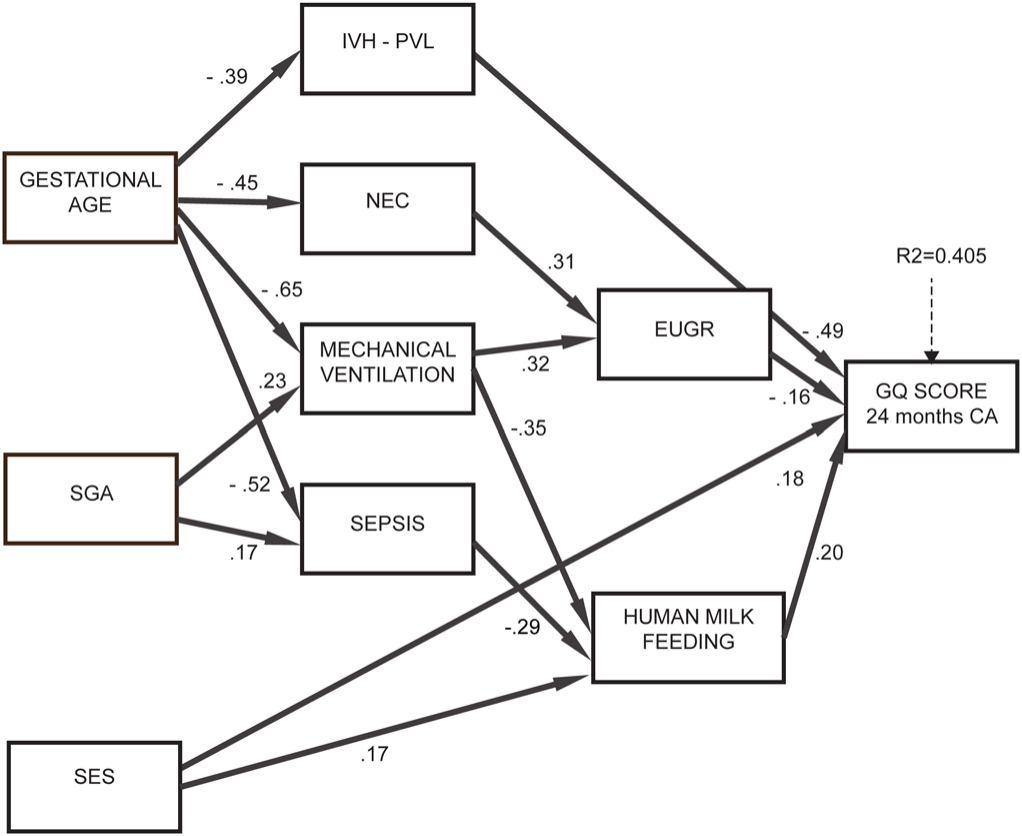
Results of path analysis, model 2. Significant effects of GA and SGA on neurodevelopment were identified: these were mediated by human milk feeding, complications and growth restriction at discharge. Specifically, SGA newborns were more likely to have sepsis and MV and those with lower GA were more likely to have IVH/PVL, sepsis, MV and NEC; in turn, sepsis, NEC and MV were associated with EUGR. This indicates that being SGA or having a lower GA does not have a negative impact on neurodevelopment *per se*, but only when it is followed by complications. Human milk feeding was more likely in newborns with higher GA (β = 0.39, p<0.001), not SGA (β = -0.16, p = 0.026) and higher SES (β = 0.17, p = 0.013). Overall this model explained 41.2% of variance of neurodevelopment (about 10% higher than the multiple regression model) and had a satisfactory goodness of fit to the data (RMSEA = 0.036, CFI = 0.960, TLI = 0.936).

The PA models were tested on the full dataset of 316 newborns and are depicted in Figs. 1 and 2 showing all significant direct relationships among variables after trimming non significant relations. The first model (Fig. 1), in which complications and type of feeding were unrelated, confirmed the positive effect of human milk (β = 0.18, p = 0.011) and of SES (β = 0.19, p<0.001) on neurodevelopment, while IVH/PVL (β = -0.49, p<0.001) and growth restriction at discharge (β = -0.21, p = 0.13) were linked with poorer neurodevelopment. Only these four variables had a significant direct effect on GQ.

The second model (Fig. 2), in which type of feeding was assumed to be dependent on complications, had similar goodness of fit (RMSEA = 0.032, CFI = 0.966, TLI = 0.949) and a slightly lower explained variance of neurodevelopment (40.5%). Variables with a significant direct effect on GQ were the same as in Model 1: human milk feeding, EUGR, SES and IVH and/or PVL. The other complications, namely NEC, mechanical ventilation and sepsis, had no significant direct effect on GQ. Therefore, even in this model the positive effect between human milk feeding and GQ was confirmed and maintained the same magnitude (β = 0.20).

## Discussion

In this observational study we investigated the impact of human milk feeding on neurodevelopment using multiple linear regression and path analysis models and results of these models were compared. Our results indicate that human milk feeding had a positive effect on neurodevelopment, corroborating existing evidence on the effect of breastfeeding [40–42]. This effect was present after adjusting for complications, growth restriction and socio-economic status and was not mediated by these variables.

Our population had a high percentage (70.6%) of newborns fed human (34.5%) or mixed milk (36.1%) during hospitalization, as a result of a long-standing practice that encourages human milk feeding (provided as raw own mother’s milk refrigerated at 4°C for up to 24 hours) plus standard fortification. This practice was introduced in the NICU after weighing the well-known benefits of mother’s raw milk [43–50] with respect to the risk due to potential Cytomegalovirus transmission [51–53], as stated by the American Academy of Pediatrics [54]. In fact, most of the newborns who were formula fed began a human or mixed milk feeding and changed their diet during hospitalization. As a matter of fact, almost two thirds of the infants were underweight at discharge, suggesting that fortified preterm human milk, as well as preterm formula, may fail to meet the recommended energy and protein intakes [55]. Our PA model indicates that growth restriction at birth was *indirectly* related to poorer neurodevelopmental achievement. In fact, SGA newborns were more likely to have sepsis and to require mechanical ventilation. These two conditions were related to a higher probability of growth restriction at discharge. Moreover, SGA newborns’ poorer outcome was mediated by their lower probability to receive human milk feeding. Instead, growth restriction at discharge had only a *direct* effect on GQand was unrelated with type of feeding. Higher GA was *indirectly* associated with a better neurodevelopment as a result of the lower probability of experiencing complications and of the higher probability of being human milk fed.

As expected, severe IVH/PVL was strongly and *directly* associated with poorer neurodevelopmental outcomes, while the negative effect of sepsis, NEC and mechanical ventilation was mediated by growth restriction at discharge.

Socio-economic status had a positive effect on neurodevelopment that was both direct and indirect, mediated by feeding. This indicates an association between higher socio-economic status and better neurodevelopment that may depend both on the family context and on higher propension of wealthier mothers to feed their babies human milk. This finding needs further investigation, but we may argue that families with a higher SES can have a higher awareness of the benefits provided by breastfeeding and ensure preterms a better cognitive stimulation in the home environment [56].

The strengths of the study are the use of an unselected cohort of relatively large size and the accuracy of data collection. Data were gathered prospectively by trained and experienced neonatologists and psychologists in a specialized center where infants are treated with standardized and evidence-based strategies. The study center belongs to the Vermont Oxford Network and has high standards of care and morbidity and mortality rates in the best quartile compared with the benchmark [57].

The present study has some limitations. The information on duration and dosage of human milk feeding was lacking. Information on date of switching from human to formula feeding and on the onset of complications was also missing, therefore we could not establish the sequence of the events. However, to address this limitation, we tested two PA models describing different scenarios and both of them confirmed the positive association of human milk feeding with neurodevelopment. Moreover, we lost a relevant quote of newborns eligible for the study: 28.6% did not attend follow-up visits; newborns in our cohort were not representative of the general VLBW population because at birth they were on average at higher risk than those who were not followed up. Lastly, 12.7% of preterms did not attend the 24 month visit; however, because their loss to follow-up was unrelated to the outcome in the large majority of cases, their outcome was unbiasedly estimated in the path analysis model.

Future perspectives include the extension of the follow-up in order to obtain information on development at older ages.

In conclusion, we strongly encourage the use of human milk during hospitalization, because, after controlling for the effects of other biological and clinical factors, it proved to be beneficial for neurodevelopment at 24 months CA and to mitigate the negative effects of lower GA and SGA.

## Supporting Information

S1 Codebook This file reports the format and the labels of the variable in S1 Dataset. (PDF)

S1 Dataset This file includes the 316 cases and 29 variables used in the Path Analysis. (XLS)

## References

[1] Mercier CE, Dunn MS, Ferrelli KR, Howard DB, Soll RF (2010) Neurodevelopmental outcome of extremely low birth weight infants from the Vermont Oxford network: 1998–2003. Neonatology 97: 329–338. 10.1159/000260136 19940516 PMC2889257

[2] Larroque B, Ancel P-Y, Marret S, Marchand L, André M, et al (2008) Neurodevelopmental disabilities and special care of 5-year-old children born before 33 weeks of gestation (the EPIPAGE study): a longitudinal cohort study. Lancet 371: 813–820. 10.1016/S0140-6736(08)60380-3 18328928

[3] Marlow N, Wolke D, Bracewell MA (2005) Neurological and developmental disability at six years of age after extremely preterm birth. N Engl J Med 352: 9–19. 10.1056/NEJMoa041367 15635108

[4] Als H, Duffy FH, McAnulty GB, Rivkin MJ, Vajapeyam S, et al (2004) Early Experience Alters Brain Function and Structure. Pediatrics 113: 846–857. 10.1542/peds.113.4.846 15060237

[5] Ziegler EE, O’Donnell AM, Nelson SE, Fomon SJ (1976) Body composition of the reference fetus. Growth 40: 329–341. 1010389

[6] Agostoni C, Buonocore G, Carnielli VP, De Curtis M, Darmaun D, et al (2010) Enteral nutrient supply for preterm infants: commentary from the European Society of Paediatric Gastroenterology, Hepatology and Nutrition Committee on Nutrition. J Pediatr Gastroenterol Nutr 50: 85–91. 10.1097/MPG.0b013e3181adaee0 19881390

[7] Tudehope DI (2013) Human milk and the nutritional needs of preterm infants. J Pediatr 162: S17–S25. 10.1016/j.jpeds.2012.11.049 23445843

[8] Brandt I, Sticker EJ, Lentze MJ (2003) Catch-up growth of head circumference of very low birth weight, small for gestational age preterm infants and mental development to adulthood. J Pediatr 5: 463–468. 10.1067/mpd.2003.149 12756374

[9] Morgan JA, Young L, McCormick FM, McGuire W (2012) Promoting growth for preterm infants following hospital discharge. Arch Dis Child Fetal Neonatal Ed 97: F295–F298. 10.1136/adc.2009.170910 21406452

[10] O’Connor DL, Khan S, Weishuhn K, Vaughan J, Jefferies A, et al (2008) Growth and nutrient intakes of human milk-fed preterm infants provided with extra energy and nutrients after hospital discharge. Pediatrics 121: 766–776. 10.1542/peds.2007-0054 18381542

[11] McCormick FM, Henderson G, Fahey T, McGuire W (2010) Multinutrient fortification of human breast milk for preterm infants following hospital discharge. Cochrane Database Syst Rev: CD004866 20614438 10.1002/14651858.CD004866.pub3

[12] Giannì ML, Roggero P, Amato O, Picciolini O, Piemontese P, et al (2014) Randomized outcome trial of nutrient-enriched formula and neurodevelopment outcome in preterm infants. BMC Pediatr 14: 74 10.1186/1471-2431-14-74 24645671 PMC3994650

[13] De Curtis M, Rigo J (2004) Extrauterine growth restriction in very‐low‐birthweight infants. Acta Paediatr 93: 1563–1568. 10.1111/j.1651-2227.2004.tb00844.x 15841762

[14] Ehrenkranz RA, Dusick AM, Vohr BR, Wright LL, Wrage LA, et al (2006) Growth in the neonatal intensive care unit influences neurodevelopmental and growth outcomes of extremely low birth weight infants. Pediatrics 117: 1253–1261. 10.1542/peds.2005-1368 16585322

[15] Cooke R (2011) Nutrition of preterm infants after discharge. Ann Nutr Metab 58: 32–36. 10.1159/000323385 21701165

[16] Cooke RWI, Foulder-Hughes L (2003) Growth impairment in the very preterm and cognitive and motor performance at 7 years. Arch Dis Child 88: 482–487. 10.1136/adc.88.6.482 12765911 PMC1763118

[17] Cooke RWI (2006) Are there critical periods for brain growth in children born preterm? Arch Dis Child Fetal Neonatal Ed 91: F17–F20. 10.1136/adc.2005.077438 16223756 PMC2672640

[18] Rozé J-C, Darmaun D, Boquien C-Y, Flamant C, Picaud J-C, et al (2012) The apparent breastfeeding paradox in very preterm infants: relationship between breast feeding, early weight gain and neurodevelopment based on results from two cohorts, EPIPAGE and LIFT. BMJ Open 2: e000834 10.1136/bmjopen-2012-000834 22492388 PMC3323805

[19] Schanler RJ, Shulman RJ, Lau C (1999) Feeding strategies for premature infants: beneficial outcomes of feeding fortified human milk versus preterm formula. 10.1542/peds.103.6.1150 10353922

[20] Griffiths R (1996) The Griffiths mental development scales from birth to two years, manual, the 1996 revision.

[21] Johnson S, Marlow N (2006) Developmental screen or developmental testing? Early Hum Dev 82: 173–183. 10.1016/j.earlhumdev.2006.01.008 16504424

[22] Giannì ML, Picciolini O, Vegni C, Gardon L, Fumagalli M, et al (2007) Twelve-month neurofunctional assessment and cognitive performance at 36 months of age in extremely low birth weight infants. Pediatrics 120: 1012–1019. 10.1542/peds.2006-3364 17974738

[23] Sansavini A, Savini S, Guarini A, Broccoli S, Alessandroni R, et al (2011) The effect of gestational age on developmental outcomes: a longitudinal study in the first 2 years of life. Child Care Health Dev 37: 26–36. 10.1111/j.1365-2214.2010.01143.x 20666779

[24] Kline RB (2011) Principles and practice of structural equation modeling. New York, NY: The Guilford Press.

[25] Aggett PJ, Agostoni C, Axelsson I, De Curtis M, Goulet O, et al (2006) Feeding preterm infants after hospital discharge: a commentary by the ESPGHAN Committee on Nutrition. J Pediatr Gastroenterol Nutr 42: 596–603. 10.1097/01.mpg.0000221915.73264.c7 16707992

[26] Bertino E, Spada E, Occhi L, Coscia A, Giuliani F, et al (2010) Neonatal anthropometric charts: the Italian neonatal study compared with other European studies. J Pediatr Gastroenterol Nutr 51: 353–361. 20601901 10.1097/MPG.0b013e3181da213e

[27] Haschke F, van’t Hof MA (2000) Euro-Growth references for breast-fed boys and girls: influence of breast-feeding and solids on growth until 36 months of age. Euro-Growth Study Group. J Pediatr Gastroenterol Nutr 31 Suppl 1: S60–S71. 10896090 10.1097/00005176-200007001-00006

[28] Papile LA, Burstein J, Burstein R, Koffler H (1978) Incidence and evolution of subependymal and intraventricular hemorrhage: a study of infants with birth weights less than 1,500 gm. J Pediatr 92: 529–534. 10.1016/S0022-3476(78)80282-0 305471

[29] International Committee for the Classification of Retinopathy of Prematurity (2005) The international classification of retinopathy of prematurity revisited. Arch Ophthalmol 123: 991–999. 10.1001/archopht.123.7.991 16009843

[30] Hollingshead A (1975) Four factor index of social status. Yale J Sociol 8: 21–52.

[31] Ostrea EM, Reyes A, Villanueva-Uy E, Pacifico R, Benitez B, et al (2012) Fetal exposure to propoxur and abnormal child neurodevelopment at 2 years of age. Neurotoxicology 33: 669–675. 10.1016/j.neuro.2011.11.006 22155319 PMC3509383

[32] Dessardo NS, Mustać E, Dessardo S, Banac S, Peter B, et al (2012) Chorioamnionitis and chronic lung disease of prematurity: a path analysis of causality. Am J Perinatol 29: 133–140. 10.1055/s-0031-1295654 22147641

[33] Muthén L, Muthén B (2012) Mplus User’s Guide. Seventh Ed. Los Angeles CA: Muthén & Muthén.

[34] Schafer JL, Graham JW (2002) Missing data: Our view of the state of the art. Psychol Methods 7: 147–177. 10.1037/1082-989X.7.2.147 12090408

[35] Steiger JH, Lind JC (1980) Statistically based tests for the number of common factors. Annu Meet Psychom Soc.

[36] Bentler PM (1990) Comparative fit indexes in structural models. Psychol Bull 107: 238–246. 10.1037/0033-2909.107.2.238 2320703

[37] Tucker LR, Lewis C (1973) A reliability coefficient for maximum likelihood factor analysis. Psychometrika 38: 1–10. 10.1007/BF02291170

[38] Browne MW, Cudeck R (1992) Alternative Ways of Assessing Model Fit. Sociol Methods Res 21: 230–258. 10.1177/0049124192021002005

[39] Hu L, Bentler PM (1999) Cutoff criteria for fit indexes in covariance structure analysis: Conventional criteria versus new alternatives. Struct Equ Model A Multidiscip J 6: 1–55. 10.1080/10705519909540118

[40] Belfort MB, Rifas-Shiman SL, Kleinman KP, Guthrie LB, Bellinger DC, et al (2013) Infant feeding and childhood cognition at ages 3 and 7 years: Effects of breastfeeding duration and exclusivity. JAMA Pediatr 167: 836–844. 10.1001/jamapediatrics.2013.455 23896931 PMC3998659

[41] Quigley MA, Hockley C, Carson C, Kelly Y, Renfrew MJ, et al (2012) Breastfeeding is associated with improved child cognitive development: a population-based cohort study. J Pediatr 160: 25–32. 10.1016/j.jpeds.2011.06.035 21839469

[42] Bernard JY, De Agostini M, Forhan A, Alfaiate T, Bonet M, et al (2013) Breastfeeding duration and cognitive development at 2 and 3 years of age in the EDEN mother-child cohort. J Pediatr 163: 36–42.e1. 10.1016/j.jpeds.2012.11.090 23312681

[43] Corpeleijn WE, Kouwenhoven SM, Paap MC, van Vliet I, Scheerder I, et al (2012) Intake of own mother’s milk during the first days of life is associated with decreased morbidity and mortality in very low birth weight infants during the first 60 days of life. Neonatology 102: 276–281. 10.1159/000341335 22922675

[44] Meinzen-Derr J, Poindexter B, Wrage L, Morrow AL, Stoll B, et al (2009) Role of human milk in extremely low birth weight infants’ risk of necrotizing enterocolitis or death. J Perinatol 29: 57–62. 10.1038/jp.2008.117 18716628 PMC2801431

[45] Henderson G, Anthony MY, McGuire W (2007) Formula milk versus maternal breast milk for feeding preterm or low birth weight infants. Cochrane Database Syst Rev. 10.1002/14651858.CD004862.pub2 17943777

[46] Montjaux-Régis N, Cristini C, Arnaud C, Glorieux I, Vanpee M, et al (2011) Improved growth of preterm infants receiving mother’s own raw milk compared with pasteurized donor milk. Acta Paediatr Int J Paediatr 100: 1548–1554. 10.1111/j.1651-2227.2011.02389.x 21707744

[47] Vohr BR, Poindexter BB, Dusick AM, McKinley LT, Wright LL, et al (2006) Beneficial effects of breast milk in the neonatal intensive care unit on the developmental outcome of extremely low birth weight infants at 18 months of age. Pediatrics 118: e115–e123. 10.1542/peds.2005-2382 16818526

[48] Vohr BR, Poindexter BB, Dusick AM, McKinley LT, Higgins RD, et al (2007) Persistent Beneficial Effects of Breast Milk Ingested in the Neonatal Intensive Care Unit on Outcomes of Extremely Low Birth Weight Infants at 30 Months of Age. Pediatrics 120: e953–e959. 10.1542/peds.2006-3227 17908750

[49] Morley R, Fewtrell MS, Abbott RA, Stephenson T, MacFadyen U, et al (2004) Neurodevelopment in children born small for gestational age: a randomized trial of nutrient-enriched versus standard formula and comparison with a reference breastfed group. 10.1542/peds.113.3.515 14993543

[50] Isaacs EB, Fischl BR, Quinn BT, Chong WK, Gadian DG, et al (2010) Impact of breast milk on intelligence quotient, brain size, and white matter development. Pediatr Res 67: 357–362. 10.1203/PDR.0b013e3181d026da 20035247 PMC2939272

[51] Capretti MG, Lanari M, Lazzarotto T, Gabrielli L, Pignatelli S, et al (2009) Very low birth weight infants born to cytomegalovirus-seropositive mothers fed with their mother’s milk: a prospective study. J Pediatr 154: 842–848. 10.1016/j.jpeds.2008.12.046 19230896

[52] Hamprecht K, Maschmann J, Vochem M, Dietz K, Speer CP, et al (2001) Epidemiology of transmission of cytomegalovirus from mother to preterm infant by breastfeeding. Lancet 357: 513–518. 10.1016/S0140-6736(00)04043-5 11229670

[53] Lanzieri TM, Dollard SC, Josephson CD, Schmid DS, Bialek SR (2013) Breast milk-acquired cytomegalovirus infection and disease in VLBW and premature infants. Pediatrics 131: e1937–e1945. 10.1542/peds.2013-0076 23713111 PMC4850548

[54] American Academy of Pediatrics. Committee on Infectious Diseases, editor (2003) Red Book: 2003 Report of the Committee on Infectious Diseases. 26th ed. Elk Grove Village, IL: American Academy of Pediatrics.

[55] Corvaglia L, Aceti A, Paoletti V, Mariani E, Patrono D, et al (2010) Standard fortification of preterm human milk fails to meet recommended protein intake: Bedside evaluation by Near-Infrared-Reflectance-Analysis. Early Hum Dev 86: 237–240. 10.1016/j.earlhumdev.2010.04.001 20447779

[56] Potijk M, Kerstjens J, Bos A, Reijneveld S, de Winter A (2013) Developmental Delay in Moderately Preterm-Born Children with Low Socioeconomic Status: Risks Multiply. J Pediatr 163: 1289–1295. 10.1016/j.jpeds.2013.07.001 23968750

[57] Lucas A, Bishop NJ, King FJ, Cole TJ (1992) Randomised trial of nutrition for preterm infants after discharge. 10.1136/adc.67.3.324 PMC1793654 1575558

