# Supplementary material for: Positive Effect of Human Milk Feeding during NICU Hospitalization on 24 Month Neurodevelopment of Very Low Birth Weight Infants: An Italian Cohort Study
Source: PLoS One. 2015 Jan 15;10(1):e0116552. doi: 10.1371/journal.pone.0116552 (PMC4295863; doi:10.1371/journal.pone.0116552)
Supplement: S1 Codebook — (PDF) [file pone.0116552.s001.pdf]

```
Contains data from PAmilk_PlosOne.dta
  obs:           316
 vars:            29                          2 Oct 2014 12:04
 size:        24,016
-----------------------------------------------------------------------------------------------
              storage   display    value
variable name   type    format     label      variable label
-----------------------------------------------------------------------------------------------
ID              int     %9.0g
males           byte    %9.0g                 Males
GA              byte    %9.0g                 Gestational age
GA_cent         byte    %9.0g                 centered GA
wgt_birth       int     %9.0g                 Weight at birth
stdw_birth      double  %9.2f                 Std. weight at birth
SGA             byte    %9.0g                 Small for Gestational Age
IVH             byte    %9.0g                 Intraventricular haemorrhage
IVH_PVL         byte    %9.0g                 IVH and/or PVL
PVL             byte    %9.0g                 Periventricular leukomalacia
MV              byte    %9.0g                 Mechanical ventilation
BPD             byte    %9.0g                 Bronchopulmonary Dysplasia
sepsis          byte    %9.0g                 Sepsis
ROP             byte    %9.0g                 Retinopathy of Prematurity
NEC             byte    %9.0g                 Necrotizing Enterocolitis
wgt_disch       int     %9.0g                 Weight at discharge
stdw_disch      double  %9.2f                 Std. weight at discharge
EUGR            byte    %9.0g                 Growth restriction at discharge
diet_disch      byte    %9.0g      feeding    Diet at discharge
diet_d          byte    %9.0g      diet       Diet at discharge dichotomous
fortification   byte    %9.0g                 Fortification at discharge
wgt_24m         int     %9.0g                 Weight at 24 months
stdw_24m        double  %9.2f                 Std. weight at 24 months
growth_24m      byte    %9.0g                 Growth restriction at 24 months CA
GQ_24m          int     %9.0g                 Neurodevelopment at 24 months
GQ_24m_tr       double  %9.0g                 GQ24 centered and scaled for * 0.1
GQ_24m_cl       byte    %9.0g                 Classes of neurodevelopment at 24 months
SES             double  %9.0g                 Hollingshead Index
SES_tr          double  %9.0g                 Hollingshead Index centered and scaled for * 0.1
-----------------------------------------------------------------------------------------------
```
